# Embedding cognitive framework with self-attention for interpretable knowledge tracing

**DOI:** 10.1038/s41598-022-22539-9

**Published:** 2022-10-20

**Authors:** Yanjun Pu, Wenjun Wu, Tianhao Peng, Fang Liu, Yu Liang, Xin Yu, Ruibo Chen, Pu Feng

**Affiliations:** 1 School of Computer Science and Engineering, Beihang University, Beijing, 100191 China; 2 Institute of Artificial Intelligence, Beihang University, Beijing, 100191 China

**Keywords:** Electrical and electronic engineering, Information technology

## Abstract

Recently, deep neural network-based cognitive models such as deep knowledge tracing have been introduced into the field of learning analytics and educational data mining. Despite an accurate predictive performance of such models, it is challenging to interpret their behaviors and obtain an intuitive insight into latent student learning status. To address these challenges, this paper proposes a new learner modeling framework named the EAKT, which embeds a structured cognitive model into a transformer. In this way, the EAKT not only can achieve an excellent prediction result of learning outcome but also can depict students’ knowledge state on a multi-dimensional *knowledge component*(KC) level. By performing the fine-grained analysis of the student learning process, the proposed framework provides better explanatory learner models for designing and implementing intelligent tutoring systems. The proposed EAKT is verified by experiments. The performance experiments show that the EAKT can better predict the future performance of student learning(more than 2.6% higher than the baseline method on two of three real-world datasets). The interpretability experiments demonstrate that the student knowledge state obtained by EAKT is closer to ground truth than other models, which means EAKT can more accurately trace changes in the students’ knowledge state.

## Introduction

Over the past decade, the *Intelligent tutoring system* (ITS) has become increasingly important in online education because it can offer a personalized and adaptive learning experience for a large scale of students. The core component of the ITS is a cognitive learner model, which can infer the latent knowledge state of individual students so that other components can provide personalized guidance for improving their learning efficiency^1–3^. In recent years, many knowledge tracing models have been developed, and they can be roughly divided into structured knowledge tracing models and deep knowledge tracing models^4^. A *Knowledge Tracing*(KT) task can be regarded as a supervised sequence learning problem. For instance, for a given sequence of a student’s historical exercise interactions $X_t$ = ($x_1$, $x_2$,......,$x_t$), the KT model can predict the probability of answering correctly in the next interaction $p(r_{t+1}=1 | q_{t+1}, X)$ and infer a student’s knowledge state in each interaction. Input $x_t$ is usually represented as a tuple ($q_t$, $a_t$), where $q_t$ represents the question that a student encounters in timestamp *t*, and $a_t$ indicates whether the answer of $q_t$ is correct or not. Structured knowledge tracing methods, such as *Bayesian knowledge tracing* (BKT)^5^, define their parameters and variables based on the principles of cognitive and education science. Therefore, it is simple to interpret their predictive results to instructors when they are used to assess a student’s learning performance. Traditional structured models have certain limitations in modeling multi-dimensional knowledge states because they typically assume that every item $q_t$ is designed around a single knowledge concept. However, an item $q_t$ involves multiple skill requirements, which can be formulated as a Q-matrix^6^, which is a sparse matrix for measurement of cognitive mastery. As an extension of BKT, an *Automatic Temporal Cognitive* (ATC) method integrates the Q-matrix into a nonlinear state-space model to trace multi-dimensional student knowledge states accurately^7^.

Recently, deep neural network-based cognitive learner models have been proposed to solve the KT tasks, such as *Deep Knowledge Tracing* (DKT)^8^, *Dynamic Key-Value Memory Network* (DKVMN)^9^, *Exercise-aware Knowledge Tracing* (EKT)^10^, and Deep-IRT^11^. These models adopt different DNN frameworks to realize knowledge tracing, and compared to structured models, they can achieve better prediction performance. However, deep neural networks are often deemed as black-box models whose complex inner representations are difficult to associate with an explicit description of latent skill states and their relations in the Q-matrix form. The DKT dismisses information about concepts and abstracts the students’ ability as a hidden vector, resulting in the invisibility of students’ cognitive structure. In addition, a number of deep learning-based learner models, such as DKVMN and EKT, assume that each question is related only to one skill without considering the difficulty coefficient of questions. Researchers have attempted to apply the IRT model to the KT tasks and combined it with the DKVMN to develop a deep-IRT model, assigning neural network parameters with psychological significance. However, a simple IRT model cannot accurately describe the knowledge requirements of exercises in complex multi-skill learning scenarios^12^. Although the Deep-IRT adds difficulty attributes to every *Knowledge Component*(KC), it still follows the assumption of a single KC for each question.

Although structured knowledge tracing models have explainable parameters based on cognitive science theories, they show certain difficulties in handling complex model structures and datasets. In contrast, deep knowledge-based tracing models can achieve good predictive performance, but their encoding of the cognitive state is very hard to interpret in the context of intelligent tutoring. To address this challenge, this study proposes the EAKT model by incorporating the cognitive structure of a structured ATC model into a DNN-based knowledge tracing framework. In this way, the predictive power of the DNN-based knowledge tracing models is combined with the strength of structured models to generate an interpretable knowledge state.

The major contributions of this paper can be summarized as follows:

1. A structured cognitive model is used to constrain a DNN-based knowledge tracing framework so that the model parameters can be assigned with explainable meanings while guaranteeing the predictive performance;
2. A Q-matrix is introduced to describe the fine-grained relationship between knowledge components and every question. The EAKT represents the multi-dimensional KC vector as a student’s knowledge state and the Q-matrix as a skill requirement for every question.

The rest of this paper is organized as follows. The related work on the Q-matrix discovery method and KT task, including Bayesian KT models and deep learning-based KT models are discussed in “Related work” section. Section “Proposed model” describes the structure of the EAKT model in detail. In “Implementation and experimental results” section, we presents the implementation details,experimental results and compares the EAKT’s performance with that of the state-of-art deep knowledge tracing models. Last section concludes this work and presents future work directions.

## Related work

### Structured knowledge tracing model

The BKT model tracks students’ knowledge states over time using the *Hidden Markov Model* (HMM). However, it can track only students’ mastery of a single cognitive skill without specifying the difficulty of learning items. Recent research efforts on the BKT have focused on the multiple-subskill extension of the BKT. Brenes^13^ proposed Dynamic Cognitive Tracing to construct a cognitive model and a student model of longitudinal student data. In his later work, he introduced the *Feature-Aware Student Knowledge Tracing* (FAST)^14^ to different incorporate skill features such as subskills and used problem’s difficulty and student ability as parameters of the KT model. All these feature-based extensions of the BKT strongly rely on experts’ knowledge when predefining the skill and subskill features without using any automatic Q-matrix discovery method. The *Automatic Temporal Cognitive Model* (ATC) represents an evolution of the *Cognitive Diagnosis Model*(CDM) and the KT Model. It aims to incorporate the multi-dimensional knowledge state and temporal changes, including skill enhancement and forgetting factors. In the ATC model, a nonlinear state-space framework is used to encode multi-dimensional KC levels and Q-matrix of learning items. The ATC model is also capable of deriving the detailed values of the Q-matrix from student learning trajectories in a data-driven approach. Therefore, the ATC model could be an ideal candidate for governing deep neural networks for knowledge tracing and improving their interpretability.

### DNN-based knowledge tracing models

#### DKT and its extensions

*Deep knowledge tracing* (DKT) uses *Recurrent Neural Networks* (RNNs) to model student learning and achieves impressive predictive advantages without the need for human-engineered features, such as recency effect and contextualized trial sequence. However, latent encoding of the knowledge state in the DKT cannot consistently depict students’ mastery of KCs and predict temporal changes in knowledge state across time. Aiming to the DKT’s major problems in KC modeling, the DKT+ introduces regularization terms, which correspond to the reconstruction and waviness, to the loss function of the original DKT model to enhance the consistency in prediction. Experiments have shown that the regularized loss function can effectively alleviate the two problems without degrading the original task of DKT^15^. Chen^16^ aimed to address the data sparse problem by incorporating the prerequisite concept pairs as constraints in the DKT model, thus improving the prediction performance of students’ concept mastery and offering a partial interpretation of predictive results. However, despite these advantages, hidden state variables of a neural network cannot explicitly represent explainable educational meanings without inducing prior cognitive structure and constraints. As a result, how to characterize changes in the students’ knowledge state accurately using a deep neural network has still been a challenge.

#### Deep knowledge tracing with attention mechanism

Attention mechanism^17^ has been shown to be effective in tasks involving sequence modeling. The idea behind this mechanism is to focus on relevant elements of the input signals when predicting the output. The *self-attentive knowledge tracing* (SAKT)^18^ has been the first method to adopt attention mechanisms in the context of KT. Attention mechanisms are more flexible than recurrent and memory-based neural networks. Extensive experiments on a variety of real-world datasets suggest that the SAKT model can outperform the state-of-the-art methods and is one order of magnitude faster than the RNN-based approaches. Ghosh et al.^19^ presented a *context-aware attentive knowledge tracing* (AKT) model, incorporating the self-attention mechanism with cognitive and psychometric models. They defined context-aware representations of questions and responses using a monotonic attention mechanism to summarize every learner’s historical performance in the right time scale. They used the Rasch model to capture individual differences between questions covering the same concept. However, none of the existing methods have quantitatively analyzed the interpretability of students’ knowledge state.

Despite of the recent progress in the research of knowledge tracing, most modeling frameworks haven’t presented an interpretable mult-dimension knowledge tracing solution. Table 1 summarizes the status of the past major proposals in the research community.

**Table 1:** Comparison of the BKT, CDM, ATC,DKT/SAKT, DKVMN/Deep-IRT, AKT, EAKT frameworks.

	Multi-dimension skill in KC level	Temporal knowledge state tracing	Processing power for large datasets	Explainability of Knowledge state
BKT	No	Yes	No	Yes
CDM	Yes	No	No	Yes
ATC	Yes	Yes	No	Yes
DKT/SAKT	No	Yes	Yes	No
DKVMN/Deep-IRT	No	Yes	Yes	Yes
AKT	No	Yes	Yes	Yes
EAKT	Yes	Yes	Yes	Yes

## Proposed model

In this section, the EAKT model, which is developed based on the attentive knowledge tracing model and the skill encoding and prediction methods of the ATC model, is presented. First, the ATC model and cognitive diagnosis models for Q-matrix are briefly introduced, and then the EAKT model is described in detail.

### ATC model

The ATC framework can be described as two parts: the first part is the probability of students answering the exercises correctly, in which the students’ knowledge state and exercise KC are both represented as multi-dimensional vectors. The second part depicts the dynamic changes of students’ knowledge states by Eq. (2). The specific calculation process of Eq. (1) is as follows. First, calculate the projection length of the student’s knowledge state $\mathbf {\varvec{\theta }}_{s t}$ on the exercise KC $\mathbf {{\varvec{a}}}_{i}$ and make a difference with the norm of exercise KC, then use the logistic function to normalize the difference $q_{sit}$ between 0 and 1 as the probability of student *s* correctly answering the exercise *i*.

1 $$\begin{aligned} \begin{aligned} q_{sit}&= \frac{\mathbf {\theta }_{st} \cdot \mathbf {a}_i}{\left\| \mathbf {a}_i \right\| } -\left\| \mathbf {a}_i \right\| \\ p_{sit}&= Pr(R_{sit}=1|\mathbf {\theta }_{st}, \mathbf {l}_i,\mathbf {a}_i) = \phi (q_{sit}) \end{aligned} \end{aligned}$$

- $\mathbf {a}_i$ represents the required KC vector of an exercise *i*;
- $\mathbf {\theta }_{st}$ represents the knowledge state vector of a student *s* at time *t*;
- $R_{sit}$ is the response of a student *s* on a exercise *i* at time *t*;
- $p_{sit}$ is the probability of a student *s* giving a correct response on an exercise *i* at time *t*.

An exercise is represented as ${\mathbf {{\varvec{a}}}_{i}} = (a_{i1}, a_{i2},\ldots ,a_{ik},\ldots ,a_{iM})$, where $a_{ik}$ represents a latent KC of a Q-matrix $Q_{MN}$. Traditionally, an element $a_{ik}$ is defined as a binary value determining whether a knowledge component $KC_{k}$ associates with an exercise $a_{i}$ or not^20^. In the ATC model, the binary Q-matrix is extended to a new matrix with real numbers to indicate the degree of correlation between exercises and all KCs.

Equation (2) assumes that a student’s knowledge state at a time step $(t + 1)$ follows the Gaussian distribution with the mean $\mu _{s(t+1),n}$, which depends on the temporal change in $\theta _{st,n}$ in the previous time step. Such a state transition represents an interplay between knowledge acquisition and exponential forgetting between the two states. Equation (3) defines a nonlinear transformation function to formulate the state transition in the learning process over the exercise-answering sequence.

2 $$\begin{aligned} \theta _{s(t+1), n} \sim N\left( \mu _{s(t+1), n^{\prime }} \sigma ^{2}\right) \end{aligned}$$

3 $$\begin{aligned} \begin{aligned} \mu _{s(t+1), n}&=\left( \theta _{s t, n}+l_{i, n} * \phi \left( q_{s i t}\right) \right) * f_{s t, n} \\ f_{s t, n}&=\exp \left\{ -\left[ \frac{1}{1+\theta _{s t, n}} * r+\beta \right] * \Delta t\right\} \end{aligned} \end{aligned}$$

- $\theta _{st,n}$ indicated the ability of the *n*th skill in the dimension of $\mathbf {\theta }_{st}$;
- $$l_{i,n} $$ denotes the value of the *n*th dimension of a vector $$\mathbf {l}_{i} $$;
- *r* and $\beta$ are fitting parameters;
- $$f_{st,n} $$ is the forgetting coefficient of a student s from time *t* to time (*t* + 1);
- $\Delta _t$ is the interval between time *t* and time (*t* + 1).

### Cognitive diagnosis models for Q-matrix

The input to the EAKT model requires a Q-matrix, and there are two ways to discover the Q-matrix. In addition to the ATC model described above, the *non-negative matrix factorization* (NMF) models have been proposed to discover the Q-matrix. These factorization techniques can implicitly encode the “slip” and “guess” factors, which means “learner effect” and the “task effect”. It divides a large unit into small sections, which are further divided into small problems, and, finally, into small steps, so that tasks can be described as specific skills required to solve the problem. NMF method approximates a matrix *X* by the product of two smaller matrices *W* and *H*, where $X\approx WH^T$, and $W \in R^{U\times K}$ is a matrix where each row *u* is a vector containing the *K* latent factors describing the learner *u* and $H \in R^{I\times K}$ is a matrix where each row i is a vector containing *K* factors describing task *i*. Let $w_{uk}$ and $h_{ik}$ be the elements of *W* and *H*, respectively; then, the performance *p* of a learner *u* on a task *i* is predicted by:

4 $$\begin{aligned} {\hat{p}}_{u i}=\sum _{k=1}^{K} w_{u k} h_{i k}=\left( W H^{T}\right) _{u, i} \end{aligned}$$

Although both the ATC model and the NMF method can obtain the Q-matrix for the input of EAKT. ATC model is difficult to apply this model to scenarios with thousands of students and long exercise sequences with hundreds of problems due to using sampling for training. Such scenarios often generate large-scale datasets and complex distribution of KCs in exercises, the training process of the ATC model can be very computationally demanding. In the implementation, we use a lightweight NMF method to generate the Q-matrix, while the ATC model is embedded in the prediction layer only.

Particularly, the Q-matrix generation process includes three main steps. First, a student’s response data are pre-processed to obtain the difficulty matrix $\left[ E_{i, j}\right] _{M \times N}$ of the student exercises, which is expressed as follows:

5 $$\begin{aligned} D_{i, j}=\left\{ \begin{array}{c}1-\frac{1}{T_{i, j}}, \text{ if } T_{i j} \ge 2, \quad T_{i j} \text{ represents } \text{ the } \text{ number } \text{ of } \text{ attempts } \text{ by } \text{ student } \text{ i } \text{ on } \text{ exercise } \text{ j } \\ 0, \text{ else } \end{array}\right. \end{aligned}$$

Then, the matrix D is decomposed using the NMF method in Eq. (6).

6 $$\begin{aligned} \begin{aligned} E_{M \times N}&\approx W_{M \times K} \times H_{K \times N}=\widehat{\mathrm {E}}_{\mathrm {M} \times \mathrm {N}} \\ W_{M \times K}&\ge 0, H_{K \times N} \ge 0 \end{aligned} \end{aligned}$$

Eventually, considering that there may be large similarities among the candidate KCs, the obtained matrix $U_{M \times K}$ need to be merged by a standard K-means clustering operation to construct the final Q-matrix.

### EAKT model

**Figure 1:**
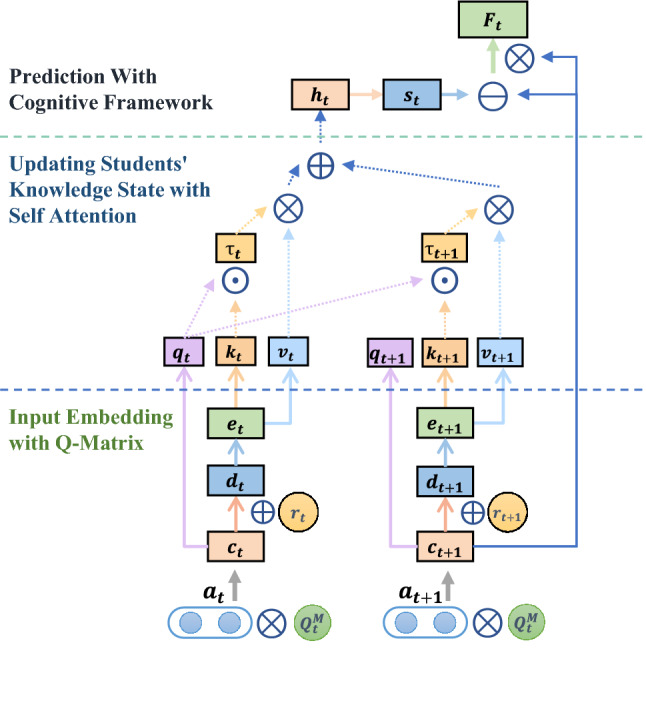
The overall structure of the EAKT model, including three parts: input embedding with the Q-matrix, updating students’ knowledge states using the attention mechanism, and making predictions by the multi-KC cognitive framework.

Recent studies have demonstrated that the DNN-based knowledge tracing models have higher prediction performance than the Bayesian-based models. Particularly, a transformer with a self-attention mechanism can significantly enhance psychometric models in characterizing changes and interrelations of complex students’ knowledge states. Therefore, it is the best choice to capture the complexity of knowledge acquisition and development as defined in Eq. (1) of the ATC framework. In view of that, the EAKT model that embeds cognitive framework of the ATC into the transformer structure is proposed. This design combines the benefits of the transformer’s supreme predictive powers for the sequential learning process and the ATC’s interpretability. The operating mechanism of the EAKT model is presented in Fig. 1, where it can be seen that it includes input embedding, knowledge state updating, and response prediction.

The workflow of the EAKT can be described as follows. At each timestamp, the EAKT model receives the current interaction information $x_t = (a_t,r_t)$ and updates a student’s knowledge state $s_t$, and then predicts the possibility of answering question $a_{t+1}$ correctly in the next timestamp according to the updated student state. In the implementation, it is assumed that *N* questions are related to *M* potential knowledge components, which can be formalized as an $N*M$ Q-matrix. It is worth mentioning that an element $Q_{ij}$ is a float value instead of a binary value, representing the capability requirement value of a question *i* to a knowledge component *j*. A student’s knowledge state and the requirements of questions are expressed in the form of a KC vector, whose dimensions represent the ability values related to the corresponding KC.

#### Input embedding with multi-dimensional Q-matrix

At time *t*, the model receives the input $x_t=(a_t,r_t)$, where $a_t$ is thee *N*-dimensional one-hot encoding of questions answered at the current moment, and $r_t$ is a binary variable representing the answering response to question $a_t$. First, it is needed to process $a_t$ according to the Q-matrix to obtain the KC vector $\mathbf {{c}_t}$ that represents the KC requirement of question $a_t$. Inspired by the dAFM, this study adds a single fully-connected layer to the input stage of the EAKT model instead of taking the Q-matrix as a fixed constant. Input layer weight is initialized by the Q-matrix and is constantly adjusted during the model training. The KC vector of the question requirement $\mathbf {c_t}$ is expressed by:

7 $$\begin{aligned} \mathbf {{c}_t} = a_t \cdot Q_t^M \end{aligned}$$

where $Q^M$ represents the weight matrix of the fully-connected layer, which is initialized by the Q-matrix obtained in advance.

Considering that the answer result has a certain influence on the change in a student’s knowledge state, the consistent operation of the DKT and its variants is to extend the one-hot encoding $a_t$ to a 2*N*-dimensional vector as an input to the RNN. However, different requirements of $a_t$ for KCs can affect the change in a student’s knowledge state with each KC. Therefore, instead of using $a_t$, this study extends $c_t$ to a a 2*M*-dimensional vector $d_t$ according to the value of $r_t$ as follows:

8 $$\begin{aligned} d_{t}=\left\{ \begin{aligned}{}[\mathbf {{c}_t} \oplus {{\textbf {0}}} ]&,&r_t=1, \\ [{{\textbf {0}}} \oplus \mathbf {{c}_t} ]&,&r_t=0. \end{aligned} \right. \end{aligned}$$

where $\oplus$ is the operation that concatenates two vectors, and ${{\textbf {0}}}$ is a zero vector in the *M* dimension; $\mathbf {{d}_t}$ goes through a fully-connected layer to generate $e_t$, which represents a neural network input, so that the network can encode more information about the interaction at time *t*.

#### Updating student knowledge state by attention mechanism

Define ${{\textbf {D}}}=(d_1,d_2,\ldots ,d_l)$, ${{\textbf {C}}}=(c_1,c_2,\ldots ,c_l)$, ${\mathbf {D}} \in {\mathbb {R}}^{2M \times l}$, and ${\mathbf {C}} \in {\mathbb {R}}^{M \times l}$, where *M* denotes the knowledge component dimension, and *l* represents the input sequence length. The query, key, and value can be respectively calculated by:

9 $$\begin{aligned} {\mathbf {Q}}={\mathbf {C}} {\mathbf {W}}^{Q}, {\mathbf {K}}={\mathbf {D}} {\mathbf {W}}^{K}, {\mathbf {V}}={\mathbf {D}} {\mathbf {W}}^{V} \end{aligned}$$

Then, the scaled dot product^21^ is used to generate ${\mathbf {H}}$ by Eq. (10), where $h_t$ is a row *t* of ${\mathbf {H}}$.

10 $$\begin{aligned} {\mathbf {H}}= \text{ Attention } ({\mathbf {Q}}, {\mathbf {K}}, {\mathbf {V}})={\text {softmax}}\left( \frac{{\mathbf {Q}} {\mathbf {K}}^{T}}{\sqrt{d}}\right) {\mathbf {V}} \end{aligned}$$

The dimension parameter is data-driven and determined by the training goal, which is higher prediction accuracy. This means the size of a hidden state $h_t$ is not directly related to the students’ knowledge states.

**Figure 2:**
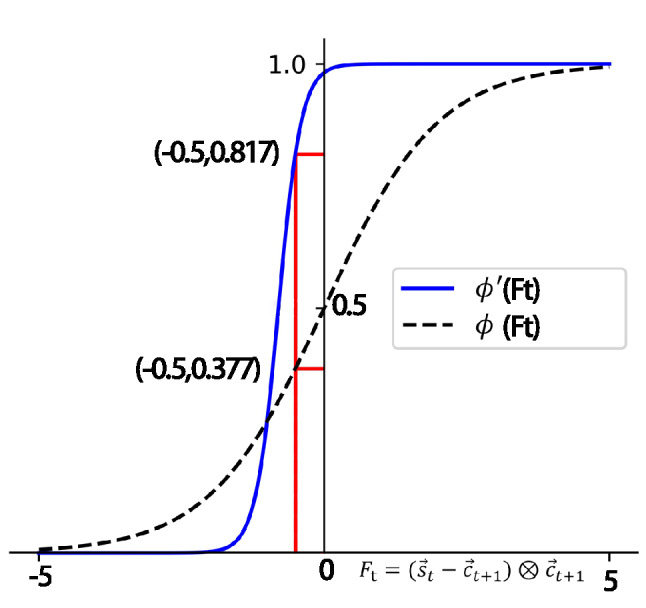
Comparison results of the two sigmoid functions.

#### Multi-KC cognitive framework prediction effect

11 $$\begin{aligned} \begin{aligned} \mathbf {c}_{t+1}&= a_{t+1} \cdot Q_t^M \\ F_t&= (\mathbf {s}_t- \mathbf {c}_{t+1}) \otimes \mathbf {c}_{t+1} \\ p_{t}&= Pr(R_{t}=1|\mathbf {s}_t, \mathbf {c}_{t+1}) = \phi ^{\prime }(F_t) \end{aligned} \end{aligned}$$

where $\otimes$ represents the element-wise multiplication operation.

Equation (11) specifies a three-step calculation. First, $s_t$ is subtracted from $\mathbf {c}_{t+1}$ to compute the difference between a student’s knowledge state and a KC dimension of questions, which is denoted as a KC difference. The measurement result of the KC difference directly affects the answering result. Second, $\mathbf {c}_{t+1}$ is set as a weight of the KC difference, and each element is multiplied to obtain the synthesis vector $F_t$. Third, the Sigmoid activation function Eq. (12) is modified to $\phi ^{\prime }$ given by Eq. (13) to adapt the structured model. This adjustment introduces a constant value *m* and a coefficient *k*; *m* is a hyperparameter, which is usually set empirically to approximately 6.9 to ensure that the student’s response probability sigmoid ($F_t$) equals one when $F_t$ equals zero. Because at that point, a student’s mastery of knowledge state should satisfy the skill requirements of the question and be able to give a correct answer for a certainty, *k* is set for adjusting the slope of the sigmoid function and empirically set to 10 for better experimental performance.

12 $$\begin{aligned} {\phi }(\mathrm {F_t})=1 /\left( 1+e^{-F_t}\right) \end{aligned}$$

13 $$\begin{aligned} {\phi ^{\prime }}(\mathrm {F_t})=1 /\left( 1+e^{-m-k*F_t}\right) \end{aligned}$$

The difference between the original sigmoid activation function and the modified activation function is presented in Fig. 2. The effectiveness of this adjustment is verified by experiments.

Overall, we provide a pseudo code in algorithm 1 of the EAKT framework to explain the EAKT model better.
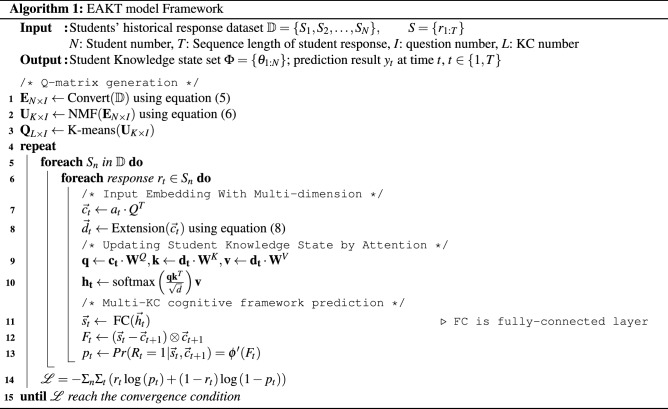


## Implementation and experimental results

### Implementation details

#### Computing infrastructure and framework setting

We now specify the network initializations in EAKT model. The model dimension in attention as 128 and the maximum allowed sequence length *l* as 50. The model is trained with a mini-batch size of five. We use Adam optimizer with a learning rate of 0.001. The dropout rate is set to 0.1 to reduce overfitting. The L2 weight decay is set to 0.000001. All the model parameters unless otherwise specified are normally initialized with 0. All the experiments are conducted on 2 * Tesla V100 PCIe 32GB GPUs. Other configuration includes 2 * Intel Xeon Gold 6148 CPU, 128GB DDR4 RAM and 1* 1024GB SATA SSD. Software environment is python 3.7.4 and pytorch 1.7.1.

### Evaluation methodology

#### Metrics

The prediction task is considered in a binary classification setting i.e., answering an exercise correctly or not. Hence, we compare the prediction performance using the *Area Under Curve* (AUC) metric. The cross entropy loss is also presented in the experimental results to reflect the degree of convergence of the model. To verify the interpretability of students’ knowledge state, we used *Word Mover’s Distance* (WMD) and *Word Rotator’ Similarity* (WRS) to compare the similarity of knowledge state between ground-truth and and obtained form different models.

#### Network training

The objective of training is to minimize the negative log likelihood of the observed sequence of student responses under the model. The parameters are learned by minimizing the cross entropy loss between $\phi ^{\prime }(\mathrm {F_t})$ and $r_t$.

14 $$\begin{aligned} {\mathcal {L}}=-\Sigma _t\left( r_t \log \left( p_t\right) +\left( 1-r_t\right) \log \left( 1-p_t\right) \right) \end{aligned}$$

### Datasets

Three publicly accessible datasets were used to evaluate the prediction accuracy of the EAKT model, and a simulated dataset was used to verify the interpretability of the EAKT model. The EAKT-Q and EAKT-P represented variants of the EAKT model, of which the former denoted a model with the Q-matrix embedding but without the cognitive framework, and the latter was a model without the Q-matrix embedding but with the cognitive framework. The statistical information of the datasets is given in Table 2. To avoid the problem of sparse inputs, the common practice of the majority of the DKT-related studies to initialize the number of KCs to the total number of problems in the ASSISTments datasets was adopted. The first dataset was ASSISTments2009 [https://sites.google.com/site/assistmentsdata/home/2009-2010-assistment-data], which was obtained by the ASSISTments online tutoring platform, contained 325k rows of responses of 4151 students answering 110 questions. The second dataset was ASSISTment2015 [https://sites.google.com/site/assistmentsdata/datasets/2015-assistments-skill-builder-data], which was obtained by the same platform, consisted of 2014–2015 school years’ student response records, containing 708k rows of non-repeating records. The records were generated by 19,917 students answering questions involving 100 questions. The third dataset was ASSISTment2017 [https://sites.google.com/view/assistmentsdatamining/dataset] containing 942,816 interactions, 1709 students, and 102 questions. These three real datasets have been widely used to evaluate the performance of the DKT and its variants.

**Table 2:** Overview of the experimental datasets.

Dataset	Student	Question	Interaction	Average length	Length variance	Maximum length
ASSIST2009	4,151	110	325,637	78	24293.4	1261
ASSIST2015	19,917	100	708,631	35	2542.6	632
ASSIST2017	1,709	102	942,816	551	175529.1	3057
Simu	20,000	30	1,000,000	50	0	50

To create a simulated dataset, a simulator with the ATC model was developed. It included a group of student agents interacting with 30 hypothetical questions and 10 KCs. In the beginning, the simulator randomly generated the initial state and a question list for each student. Then, at each simulation timestamp, it selected a question from the list to be answered for each student. Once a student solved the problem correctly, the simulator removed the problem from the student’s question list. Furthermore, the simulator updated each student’s knowledge state based on the student’s response result after each simulation iteration by:

15 $$\begin{aligned} s_t(k) = max((s_{t-1}(k)+l_k)*exp(-\beta -r*\Delta t),0) \end{aligned}$$

- $s_t(k)$ denotes the ability of a student *s* for a knowledge component *k* at time *t*;
- $l_k$ denotes the improvement in students’ ability for a knowledge component *k* after answering questions at time $(t - 1)$;
- $\beta$ and *r* are the forgetting parameters;
- $\Delta t$ denotes the time difference between the time *t* and time $(t - 1)$.

Based on the students’ knowledge state at moment *t* and the KC requirement of a question *i*, the probability of answering the question correctly was obtained by the ATC model defined by Eq. (1).

### Student performance prediction

Experimental results on all datasets are presented in Table 3. The AUC and loss values of all models were calculated to evaluate their prediction performances in the experiment. To verify the prediction accuracy of student abilities, the training and test datasets of the experiment were defined as follows. The first 80% of each student’s answer sequence was set as a training dataset, and the remaining 20% of data denoted the test dataset. There are usually two approaches to divide the test dataset, one according to the student cut and one according to the sequence cut, because in the experiment we need to verify the prediction performance of the model in addition to verify the prediction performance on the knowledge state, and the sequence cut according to the sequence can allow the previous sequence of each student to participate in the training, so that the prediction of the knowledge state is more accurate. In the experiment of the EAKT model, the KC number values of the three real datasets were 30, 10, and 30, and that of the simulated dataset was 10. The Q-matrix of the simulated dataset was generated by the ATC model. According to the comparison experiment on the simulated dataset, the Q-matrix generated by the ATC model could improve the prediction accuracy of the model. Since the ATC model cannot handle large-scale datasets, the Q-matrix of the three real datasets was obtained by the NMF.

The AUC and loss of the EAKT were compared with those of the DKT, SAKT, EAKT-Q and EAKT-P models on four datasets. For the Assist2009 dataset, the average AUC value achieved by the EAKT model was 84.6%, which was higher than those four models. The predictive performances of the five models on the ASSIST2015 dataset were similar to those on the ASSIST2009 dataset. The AUC values of the three models were 70.2%, 74.1%, 78.7%,75.0%, and 80.0%. The results indicated that the EAKT model outperformed the DKT model based the LSTM structure. The EAKT model also outperformed the SAKT model with the transformer structure on three datasets. Experimental results showed that embedding cognitive structures in a DNN-based knowledge tracing framework could improve prediction performance.

**Table 3:** Comparison of experimental results.

Method	DKT			SAKT			EAKT-Q			EAKT-P			EAKT		
	KC.num	AUC	Loss	KC.num	AUC	Loss	KC.num	AUC	Loss	KC.num	AUC	Loss	KC.num	AUC	Loss
ASSIST2009	30	81.4	0.31	30	79.9	0.32	30	84.0	1.47	30	84.0	1.61	30	**84.6**	1.48
ASSIST2015	10	70.2	2.39	10	74.1	1.66	10	78.7	5.05	10	75.0	5.35	10	**80.0**	5.62
ASSIST2017	10	**71.8**	10.03	10	66.6	10.30	10	69.0	1.57	10	69.5	2.93	10	69.5	1.54
Simu	10	81.7	20.39	10	90.0	5.67	10	90.3	4.72	10	90.0	4.60	10	**90.5**	4.59

Significant values are in [bold].

### Interpretable knowledge state

The main contribution of the EAKT model is the ability to present every student’s knowledge state in an explanatory way, which is vital for implementing adaptive personalized learning. The DKT model abstracts knowledge state representation in hidden states of an RNN, resulting in the difficulty in interpreting every student’s cognitive skill level and dynamic state changes. The EAKT model constrains the transformer by embedding the major elements of the ATC model to reveal the latent knowledge state and the changing trend over time from the hidden state of the neural network. To verify that the students’ knowledge states obtained by the EAKT model are explicable and accurate, the simulated dataset was used to compare the output result of the EAKT with the ground-truth state of each student agent at each timestamp. Two evaluation metrics, namely the word mover’s distance^22^ and word rotator’s distance^23^ were employed to calculate the similarity between the students’ latent state and inferred $s_t$ from the hidden units of the DKT, SAKT, and EAKT. Assume that a student *S* has a knowledge state sequence of ${\varvec{w}}_{1}, {\varvec{w}}_{2}, \ldots , {\varvec{w}}_{n}$, then a student $S^{\prime }$ has the knowledge state sequence ${\varvec{w}}_{1}^{\prime }, {\varvec{w}}_{2}^{\prime }, \ldots , {\varvec{w}}_{m}^{\prime }$.

1. Word mover’s distance: $p_{i}$ and $q_{j}$ are defined by Eq. (16):
  16 $$\begin{aligned} \begin{aligned} p_{i} \equiv \frac{1}{n}, q_{j}~\equiv \frac{1}{m} \end{aligned} \end{aligned}$$
  The word mover’s distance was defined by:
  17 $$\begin{aligned} \begin{aligned} {\text {WMD}}\left( S, S^{\prime }\right)&=\min _{\gamma _{i, j} \ge 0} \sum _{i, j} \gamma _{i, j}\left\| {\varvec{w}}_{i}-{\varvec{w}}_{j}^{\prime }\right\| \\ \quad \text{ s.t. } \quad \sum _{j} \gamma _{i, j}&=\frac{1}{n}, \sum _{i} \gamma _{i, j}=\frac{1}{m} \end{aligned} \end{aligned}$$
2. Word rotator’s distance: $p_{i}$ and $q_{j}$ were calculated by Eq. (18):
  18 $$\begin{aligned} \begin{aligned} p_{i}&=\frac{\left\| {\varvec{w}}_{i}\right\| }{Z}, \quad Z =\sum _{i=1}^{n}\left\| {\varvec{w}}_{i}\right\| \\ q_{j}&=\frac{\left\| {\varvec{w}}_{j}^{\prime }\right\| }{Z^{\prime }}, \quad Z^{\prime }=\sum _{j=1}^{n^{\prime }}\left\| {\varvec{w}}_{j}^{\prime }\right\| \end{aligned} \end{aligned}$$
  The word rotator’s similarity was calculated by:
  19 $$\begin{aligned} \begin{aligned} d_{i, j}&=1-\frac{{\varvec{w}}_{i} \cdot {\varvec{w}}_{j}^{\prime }}{\left\| {\varvec{w}}_{i}\right\| \times \left\| {\varvec{w}}_{j}^{\prime }\right\| } \\ {\text {WRS}}\left( S, S^{\prime }\right)&=1 - \min _{\lambda _{i, j} \ge 0} \sum _{i, j} \lambda _{i, j} d_{i, j} \\ \quad \text{ s.t. } \quad \sum _{j} \lambda _{i, j}&=p_{i}, \quad \sum _{i} \lambda _{i, j}=q_{j} \end{aligned} \end{aligned}$$

According to the above definitions, the word mover’s distance indicates a disproportion to the state similarity, while the word rotator’s similarity means the opposite. In the experiments, 4,000 students knowledge states form test dateset obtained by the EAKT model, EAKT-O model, SAKT model, and DKT model were compared with the ground-truth states. The two evaluation metrics were calculated. The EAKT-O model represented the EAKT model with the original sigmoid activation function. The sum values of the WMD and WRS were denoted by the WMD.T and WRS.T, and their average values were denoted by WMD.A and WRS.A, respectively. They were calculated by Eq. (20), where *N* is the total number of students.

20 $$\begin{aligned} \begin{aligned} WMD.T&=\sum _{i=1}^{N} {\text {WMD}}\left( S_i, {S_i}^{\prime }\right) \qquad WMD.A=\sum _{i=1}^{N} {\text {WMD}}\left( S_i, {S_i}^{\prime }\right) /N \\ WRS.T&=\sum _{i=1}^{N} {\text {WRS}}\left( S_i, {S_i}^{\prime }\right) , \qquad WRS.A=\sum _{i=1}^{N} {\text {WRS}}\left( S_i, {S_i}^{\prime }\right) /N \end{aligned} \end{aligned}$$

**Figure 3:**
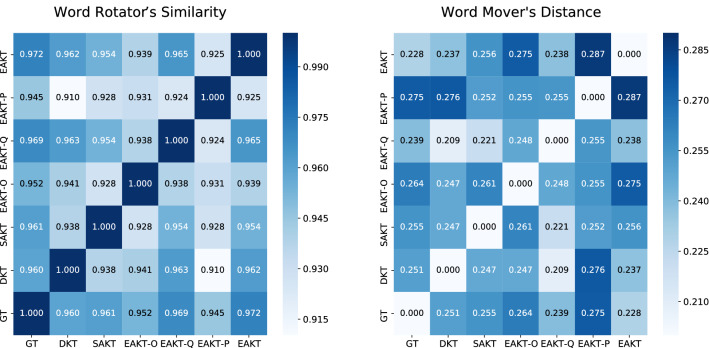
The similarity comparison of the knowledge states obtained by the four models and the ground-truth knowledge states in terms of the distance metrics.

The student’s knowledge state obtained by the EAKT model was the closest to the ground-truth state, having the highest average value of WRS.A, which was higher than those of the SAKT, EAKT-O, EAKT-Q, EAKT-P, and DKT models. The similarity matrices between the four models and the ground truth for the two distance metrics are presented in Fig. 3. The results indicated that the student’s knowledge state obtained by the EAKT model outperformed those of the other models. Interestingly, the EAKT-O performed the worst in terms of the two similarity metrics, which confirmed that the optimized activation function was effective in computing the student’s knowledge state (Supplementary information).

## Conclusions and future work

In this paper, a knowledge tracing model named the EAKT is developed using self-attention mechanism and a structured ATC model. The proposed model can trace the knowledge state of students in every timestamp while predicting their future performance. Particularly, this paper introduces a multi-dimensional KC vector to represent students’ knowledge states and a Q-matrix to represent the KC requirements of questions in deep neural networks. The experiments on real datasets and simulated datasets verify that the proposed EAKT model can obtain an interpretable multi-dimensional sequence of students’ knowledge states on the premise of preserving the prediction power of the self-attentive transformer framework. A combination of explanatory and predictive power in the EAKT model enables the better design of intelligent tutoring applications. In the future, we plan to explore the use of deep learning frameworks to enhance cognitive models, such as using adjustable weights to represent Q-matrix and enhance it through introducing exercise texts.

## Supplementary Information

Supplementary Information.

## Data Availability

The datasets generated and analysed during the current study are available in the EAKT repository, https://github.com/ranydb/EAKT.
