# Supplementary material for: Embedding cognitive framework with self-attention for interpretable knowledge tracing
Source: Sci Rep. 2022 Oct 20;12:17536. doi: 10.1038/s41598-022-22539-9 (PMC9584970; doi:10.1038/s41598-022-22539-9)
Supplement: Supplementary file 1 — Supplementary Information. [file 41598_2022_22539_MOESM1_ESM.pdf]

Appendix

In order to make the paper better read by people who are not in the field, we show the definitions and explanations of some professional abbreviations in table 4.

Abbreviation	Definition	Explanation
ITS	Intelligent tutoring system	A computer system that aims to provide immediate and customized instruction or feedback to learners
ATC	Automatic temporal cognitive	A method that integrates knowledge tracing and cognitive diagnosis
IRT	Item response theory	A theory to explain the relationship between latent traits and their manifestations
DKT	Deep knowledge tracing	A method for knowledge tracing through deep neural networks
Q-matrix	Matrix	The mapping between items and skills
LSTM	Long short-term memory	An artificial neural network used in the fields of sequences
AUC	Area under the curve	A performance measure for binary classifiers

Table 4. Abbreviations with definitions and explanation
